# Autologous Stem Cell Transplantation for Aggressive Lymphomas

**DOI:** 10.4084/MJHID.2012.075

**Published:** 2012-11-07

**Authors:** Giuseppe Visani, Paola Picardi, Patrizia Tosi, Roberta Gonella, Federica Loscocco, Teresa Ricciardi, Lara Malerba, Barbara Guiducci, Simona Tomassetti, Sara Barulli, Alessandro Isidori

**Affiliations:** 1 Hematology and Stem Cell Transplant Center, AORMN, Pesaro, Italy; 2 Hematology Unit, Department of Oncology and Hematology, Infermi Hospital, Rimini, Italy; 3 Internal Medicine Unit, AUO San Martino, Genova, Italy Italy

## Abstract

The role of high-dose therapy (HDT) followed by autologous stem cell transplantation (ASCT) in the treatment armamentarium of aggressive B- and T-cell non-Hodgkin lymphoma (NHL) is still a matter of debate. In the pre-Rituximab era, the PARMA study demonstrated the superiority of HDT/ASCT over conventional salvage chemotherapy in chemosensitive, relapsed patients. Subsequently, HDT/ASCT has become a standard approach for relapsed NHL. With the advent of Rituximab in the landscape of NHL, transplantation as part of first-line therapy has been challenged. However, no benefit in terms of disease-free or overall survival of HDT/ASCT over standard therapy was shown when Rituximab was added to both arms. Moreover, the superiority of HDT/ASCT over conventional salvage therapy in patients relapsing from first-line therapy including Rituximab was not confirmed. From these disappointing results, novel strategies, which can enhance the anti-lymphoma effect, at the same time reducing toxicity have been developed, with the aim of improving the outcome of HDT/ASCT in aggressive NHL. In T-cell lymphoma, few publications demonstrated that consolidation of complete remission with HDT/ASCT is safe and feasible. However, up to one-third of patients may never receive transplant, mostly due to progressive disease, and relapse still remains a major concern even after transplant.

## Introduction

Aggressive lymphomas comprise a variety of lymphoid malignancies which can benefit in various phases of the diseases from high-dose therapy (HDT) followed by autologous stem cell transplant (ASCT). Despite data suggesting that prolonged event- event free survival can be achieved with SCT combined with HDC, there are problems that may limit the utility of this approach for a broad patient population. Moreover, the development of Rituximab has generated a sort of skepticism on the usefulness of HDT/ASCT, especially Mediterr J Hematol Infect Dis 2012; 4; Open Journal System in front-line therapy. Consequently, there is an urgent need for other effective and well-tolerated approaches that will eradicate the residual disease that may persist before ASCT, thus improving outcomes for patients with this life-threatening disease. In addition, approaches with better safety profiles would allow older patients to benefit from this therapeutic option.

In this review, we will deeply discuss the data available about HDT followed by ASCT in aggressive lymphomas.

## High-Dose Therapy and Auto-SCT as Part of First-Line Therapy

On the first decade of study into autologous transplantation for the treatment of aggressive lymphoma, the focus was on the use of this approach to rescue patients after relapse or if the disease already progressed under standard chemotherapy. These encouraging results in relapsed or progressive lymphoma led to the testing of the technique as a primary therapy for the disease.

Thirteen prospective multicenter randomized studies have tried to evaluate the impact of HDT with ASCT as part of the first-line treatment for patients with diffuse large B cell lymphoma (DLBCL) (Table 1).^1^–^12^ In those trials, patients received initial induction with conventional dose chemotherapy and were then randomly assigned to consolidation with ASCT or additional doses of conventional chemotherapy. However, the results of prospective randomized trials are contradictory, with nine studies showing no difference in outcomes. On the other hand, 4 studies showed improvement in disease-free survival (DFS) and/or overall survival (OS) for the high-dose therapy arm. Of note, several problems have hampered the comparison of data coming from these studies. Inclusion criteria, time of randomization (diagnosis vs after induction therapy), intensity/number of courses of conventional therapy before ASCT, disease status at transplant were varied among different studies, making comparisons difficult. Nonetheless, these trials included different proportions of patients with dissimilar risk categories and different histological subtypes. Last but not least, all these trials were conducted in the pre-rituximab era and thus the results may not be applicable to current practice.

In order to assess the effects of such high-dose treatment on overall survival in patients with aggressive non-Hodgkin lymphoma, the Cochrane Hematological Malignancies Group performed in 2008 a meta-analysis that included data from 15 randomized controlled trials with a total of 3079 patients treated for aggressive NHL.^13^ In general, there was no evidence that HDT improves OS (HR 1.05; CI 0.92 to 1.19) or event free survival (EFS) (HR 0.92; CI 0.80 to 1.05). Nevertheless, in patients with good risk age-adjusted International Prognostic Index (aaIPI) there was some evidence for worse OS (HR 1.46; CI 1.02 to 2.09) when treated with HDT. In contrast, there was suggestive evidence that poor risk patients may benefit from HDT.

Overall, with respect to the large population included in the meta-analysis and the attempts made to minimize bias; the Authors concluded that there is no evidence for a general benefit of HDT followed by ASCT for patients with aggressive NHL as first-line treatment based on the data available at the time of writing (2008). In fact, the improvements in relapse free survival and complete remission rates did not translate into an overall survival benefit in the respective groups. However, if HDT is employed for high risk patients, there may be a benefit.

With the remarkable increase in the response rate when Rituximab (R) was added to conventional chemotherapy, for new studies comparing standard chemotherapy to HDT followed by SCT with the incorporation of R into both treatment concepts were required. Several phase II studies and phase III randomized trials investigated the role of HDT followed by ASCT for the initial treatment of DLBCL patients (Table 2).^14^–^20^ Tarella et al^14^ performed a prospective multicentre trial to evaluate the combination of rituximab and modified high-dose sequential (HDS) chemotherapy together with multiple peripheral blood progenitor cell (PBPC) support in newly diagnosed DLBCL patients with aaIPI 2-3. In this study, 93 of the 112 patients enrolled completed the planned therapy (5 early and 2 late toxic deaths). Overall, the CR rate was 80%. At a median follow-up of 48 months, the estimated 4-year OS projection was 76% (CI: 68–85%) and the 4-year EFS projection was 73% (CI: 64–81%). No significant differences in OS and EFS were observed between subgroups with germinal-center and activated B-cell phenotype. This study showed the efficacy of combining rituximab and early HDT with multiple PBPC in a subset of DLBCL patients with a dismal prognosis.

Vitolo et al.^15^ incorporated R (6 doses) in a dose-dense induction regimen (CEOPx4) coupled with a short intensification phase (MAD x2), followed by HDT (Conditioning: BEAM) and ASCT. Overall, 86% of the patients completed the treatment, with a CR rate of 82% (CI: 73–88%). With a median follow-up for censored patients of 49 months, the reported 4-year failure-free survival (FFS) rate was 73% (CI: 63, 5–82, 5%) and the 4-year OS rate was 80% (CI: 71,6–88,4%).

The GELA group^16^ combined 4 doses of R with 4 cycles of biweekly ACVBP chemotherapy in 208 high-risk DLBC patients. Responding patients (155 with CR or PR) were than addressed to HDT(BEAM) followed by ASCT. A total of 32 patients did not receive HDT/ASCT. Twenty five were withdrawn during induction therapy, 6 because of insufficient response before consolidation and one because of sudden death. With a median follow-up of 45 months, the 4-year PFS and OS were estimated at 76% (CI: 69–81%) and 78% (72–83%).

A case-control study by matching (1:1) patients treated with R with patients treated with identical chemotherapy (CEOP; ABCVP) program not given R but submitted to ASCT was also performed in both the Italian and French studies. Regardless of the limitations intrinsic to retrospective analyses, these comparisons demonstrated a marked therapeutic advantage of chemo-immunotherapy over chemotherapy alone in both PFS and OS.

The US/Canadian Intergroup trial (SWOG S9704)^17^ enrolled 370 patients aged 18–65 years with aaIPI 2 or 3 who received 5 courses of CHOP-21+/− R. Those patients achieving a CR or PR were randomized to 3 further courses of CHOP-21+R or one such course followed by HDT/ASCT. 370 patients started treatment with CHOP (52%) or R-CHOP (48%), 128 patients were then randomized to 3 additional courses of (R)-CHOP, 125 patients were randomized to 1 more course of (R)-CHOP followed by HDT/ASCT. PFS was significantly better (p=0.005) after HDT/autoSCT, but OS was not significantly different. The study was not powered, however, to show a difference in PFS (72% for patients undergoing HDT/ASCT vs. 62% for patients treated with R-CHOP-21) if the analysis was restricted to patients treated with R-CHOP.

The Italian lymphoma foundation (FIL)^18^ randomized upfront high-risk patients (aaIPI 2 or 3) aged 18–65 years with DLBCL or FL grade IIIb to receive 4 courses of R-CHOP-14 or R-(mega)CHOP-14 (cyclophosphamide escalated to 1200 mg/m2 and adriamycin to 70 mg/m^2^). Patients with CR or PR continued treatment as initially randomized with 4 courses of RCHOP-14, 2 courses of R-megaCHOP or R-MAD (mitoxanthrone, cytarabine, dexamethasone) in the absence of response, in all cases followed by BEAM/ASCT. Again, 2-year-PFS was significantly higher for patients receiving HDT/ASCT as compared to R-(mega)CHOP-14 (p =0.0128), but again OS was not significantly different.

Both the SWOG and the FIL study randomized only patients with chemosensitive disease: patients without CR or PR after (R)-chemo were not eligible for transplantation. However, while the Italian study indicate that chemosensitive patients may significantly do better with HDT/ASCT with respect to further conventional therapy also when R is a part of frontline therapy, the results of the SWOG study are not conclusive because the comparison of 8 courses of R-CHOP-21 and 5 coursed of R-CHOP-21 followed by HDT/ASCT lacks the statistical power necessary to show significant differences.

The German group^19^ randomized patients between 18 and 60 years with high-risk (IPI 2 or 3) aggressive B-cell lymphoma to receive 8 cycles of CHOE(etoposide)P-14 with 6 R or 4 cycles of MegaCHOEP with escalating doses of cyclophosphamide, etoposide, adriamycin, and prednisolone also administered with 6 R. The patients randomized were equally split in the 2 groups. 3-year-EFS and OS were not significantly different (p=0.14 and p =0.081, respectively) between the 2 groups.

The GELA^20^ group randomized patients aged 18–60 years with DLBCL in all stages to 4 courses of R-CHOP-14 or 2 courses of CEEP (cyclophosphamide, epirubicin, vindesine, and prednisolone) followed by 1 course of MC (methotrexate 3 g/m2, cytarabine 100 mg/m2 for 5 days) combined with 4 R infusions. Patients were then stratified according to PET scan to receive 3 courses of R-DHAP followed by BEAM/ASCT (PET positive) or experienced a second randomization to either 4 R-CHOP-14 or BEAM/autoSCT (PET-negative). RCHOP-14 was significantly better (p=0.03) than HDT/ASCT in terms of 3-year-EFS in all IPI subgroups including patients with aaIPI 2 or 3. OS was not significantly different. However, the interpretation of this study is complicated by introducing PET-guided restaging after R-CHOP-14 and use of an alternative chemotherapy (CEEP/MC). Even if this study showed that HDT/ASCT is no better than the combination of R and CHO(E)P-14, this message is restricted to patients who were PET-negative at restaging (chemosensitive patients?) and treatment results in both the conventional and the HDT arm seem surprisingly poor.

In conclusion, results of all these studies incorporating HDT/ASCT in the frontline therapy of aggressive B-cell lymphoma are contradictory and definitive conclusions may indeed not be drawn.

## High-Dose Therapy and Auto-SCT for Relapsed Aggressive Lymphoma

The superiority of HDT and ASCT over conventional salvage chemotherapy was demonstrated by Philip et al^21^ in 1995 in a multicenter, prospective randomized trial for relapsed aggressive non-Hodgkin lymphomas. The Parma trial established HTD/ASCT as the standard therapy in relapsing aggressive NHL patients responding to salvage therapy. This study compared 2 more courses of DHAP chemotherapy to HDT/ASCT in younger patients with relapsed mostly aggressive NHL who initially had responded to 2 cycles of DHAP. Patients who were treated with DHAP followed by HDT/ASCT had significantly better EFS than patients who were treated with salvage chemotherapy alone. The 8-years EFS rate was 36% in the HDT/ASCT group and 11% in the conventional treatment group.

Recently, the randomized CORAL study,^22^ performed in the rituximab era, confirmed the results of the PARMA study, reporting a 3-year PFS of 53% in patients receiving high-dose BEAM therapy after having obtained a response with R-ICE or R-DHAP. The CORAL study randomized younger patients with relapsed or refractory aggressive B-cell lymphoma to 3 courses of either R-DHAP or R-ICE. Results were comparable between the 2 different rituximab containing regimens (R-ICE vs R-DHAP, CR rate 63.5% and 62.8%, respectively). However, only 50% of patients were able to proceed with ASCT. Moreover, in patients relapsing more than 12 months after ASCT, prior rituximab treatment did not affect PFS. Finally chemosensitive patients receiving HDT and ASCT were randomized to maintenance with Rituximab or observation. The final report^23^ confirmed the major findings of first analysis and showed that R maintenance did not significantly improve EFS, PFS, or OS.

Due to the results reported from the randomized CORAL study which are less favorable than those reported in non-randomized study,^22^–^23^ testing effective high dose therapy schedules to increase responses and to reduce both transplant related mortality (TRM) and the incidence of secondary diseases becomes an important area of research in relapsed/refractory lymphomas.^24^,^25^ Furthermore, there is no evidence, to date, for a superior high-dose therapy regimen in the treatment of refractory or relapsed aggressive lymphomas. In fact, various conditioning regimens have been used as chemotherapy treatment before ASCT, with DFS and OS rates ranging from 34 to 60% and 26 to 46%, respectively.^26^–^36^ To date, few randomized trials comparing different conditioning regimens have been performed, and no regimen has demonstrated superiority to another. Furthermore, little is known regarding the comparative toxicity and efficacy of various HDT regimens applied in lymphomas.^26^–^36^ Advances in the HDT regimens and supportive care have reduced TRM to less than 10%. However, the commonly utilized HDT regimens have lights and shadows, and new strategies with novel drugs have been considered and tested by several Investigators.

Initial studies in relapsed aggressive NHL incorporated total body irradiation (TBI) into the conditioning regimen approach as the mainstay of therapy for chemosensitive NHL. This approach was considered reasonable and effective at the time. Tissue dose limitations, however, prohibit the use of TBI in patients who have received prior consolidative or salvage radiation after initial chemotherapy. More importantly, a significant proportion of the patients treated with ASCT subsequently developed secondary myelodysplasia or acute leukemia. Most transplant centres and groups have moved away from TBI-conditioning approaches and have concentrated on chemotherapy-based regimens.^35^–^42^

Chemotherapy-based regimens consist of 2–4 drug combinations including an alkylating agent given over 6–7 consecutive days. These combination treatments induce CRs in the 60–85% range while at 2–5 years after ASCT have resulted in 34–60% DFS and 24–46% OS rates. TRM have resulted in 3.8–17%.

Kim JG et al.^43^ have reported the results of a multicenter study aimed to evaluate the efficacy and toxicity of a combination of intravenous busulfan (Bu), cyclophosphamide (Cy) and etoposide (E) (Bu/Cy/E) as a conditioning regimen prior to autologous hematopoietic stem cell transplantation in patients with NHL. 64 patients with relapsed/refractory or high risk lymphoma were enrolled. The high-dose chemotherapy consisted of i.v. Bu (0.8 mg/kg for 3 days), CY (50 mg/kg for 2 days) and E (400 mg/m^2^ for 2 days). The median age was 43 and DLBCL was the common histological subtype. At a median of follow up of 16.4 months, 15 patients (23.4%) exhibited a relapse or progression, while 13 patients (20.3%) had died of disease. The estimated 3-year OS and PFS for all patients was 72.1 and 70%, respectively demonstrating the efficacy and tolerability of this conditioning regimen.

Our group^44^ designed an Italian multicenter phase I–II study to evaluate the safety and the efficacy of increasing doses of bendamustine (160 mg/m^2^, 180 mg/m^2^ and 200 mg/m^2^ given on days -7 and -6) coupled with fixed doses of etoposide, cytarabine and melphalan (BeEAM regimen) for resistant/relapsed lymphoma patients. The basis for testing bendamustine in a novel conditioning regimen arose from preliminary in vitro data on lymphoma cell lines, demonstrating the higher efficacy bendamustine has in combination with etoposide, cytarabine and melphalan with respect to carmustine. 28 NHL patients were enrolled in the study, which also comprises 15 Hodgkin lymphoma patients. In total, 43 patients received a median number of 6x10^6^CD34^+^cells/kg (range 2.4–15.5). All patients engrafted, with a median time to ANC>0.5x10^9^/l of 10 days. All patients fully engrafted, with a median time to achieve ANC>0.5x10^9^/L and PLT>20x10^9^/l superimposable to those of other conventional conditioning regimens (10 and 13 days, respectively). The 100-day transplant-related mortality was 0%. After a median follow-up of 30 months (last update June 2012), 32/43 patients (74%) are in complete remission, whereas 11/43 relapsed and 3/43 died. Five out of 28 NHL patients (17%), as well as 6/15 (40%) HD patients relapsed. Disease type (NHL versus HD) and disease status at transplant (chemosensitive vs chemoresistant) significantly influenced disease-free survival (p=0.01; p=0.007). Remarkably, 4/43 (9%) patients achieved the first complete remission after receiving the high-dose therapy with ASCT. We concluded that the BEAM regimen is safe and effective for heavily pretreated lymphoma patients, with 74% of patients being alive and disease-free after a median follow-up of 30 months. Further studies are warranted to confirm these preliminary results. For this reason, we are currently running a new study testing Bendamustine at 200 mg/m^2^/day over 2 days in aggressive (DLBC or grade III follicular lymphoma) B-cell lymphoma.

Nieto Y et al^45^ developed a new HDT combination of infusional gemcitabine with busulfan/melphalan for lymphoyd tumors. Gemcitabine dose was escalated by extending infusions at a fixed rate of 10 mg/m^2^/min in sequential cohorts, in daily 3- or 2-dose schedules. Each dose immediately preceded busulfan or melphalan. 133 patients were enrolled (80HL, 46 NHL, 7 MM). Primary refractory disease was present in 45% of NHL patients, and 50% of patients were PET-positive at transplant. The 2-dose schedule was better tolerated. Overall response and CR rate were 100% and 69% respectively in aggressive B-cell NHL, and 66% and 66% in T-cell NHL. After a median of follow up of 24 months, EFS and OS rates are 60 and 89% respectively (B-NHL), and 70% and 70% (T-NHL).

In parallel to novel chemotherapeutic agents, several investigators tested the safety and the efficacy of adding or incorporating radioimmunotherapy in HDT prior to ASCT.

Radioimmunotherapy is a novel type of immunotherapy that uses a linker to combine a monoclonal antibody with a certain specificity linked with a radioimmunoconjugate. The currently available agents used in lymphoma target the CD20 molecule. Two radiolabeled antibodies, iodine-131 tositumomab and yttrium-90 ibritumomab, have been approved by the US Food and Drug Administration (FDA) to treat relapsed lymphoma.

Radiation therapy has been a component of the conditioning regimens used to treat lymphoma in the past. Because lymphomas are highly sensitive to radiation, radioimmunotherapy (RIT) has been used with great success in consolidation therapy and, as a result, either as a single agent or as augmentation of HDT, as part of a conditioning regimen for ASCT. The flexibility of including RIT as part of conditioning therapy also allows it to be combined with RIC to reduce the toxic effects of HDC. In fact, RIT delivers targeted radiation to lymphoma sites protecting other tissues; therefore, it limits toxicity and allows the use of ASCT in older patients or in patients with comorbidities and decreased organ function. This treatment option replaces any concomitant loss of chemotherapy efficacy with a gain in RIT efficacy. The data so far suggest that the use of RIT in the autologous setting can improve clinical outcome with no added toxicity in these patients, whereas similar positive findings have been reported in preliminary studies of RIT combined with RIC and alloSCT in high-risk patients.

Both antibodies have been used in phase I and phase II clinical trials, in conventional standard dose or escalated high dose, to increase the therapeutic effect of high-dose therapy or to substitute TBI, with the aim of reducing relapse rate without adding toxicity to the conditioning regimens. Table 3^46^–^55^ summarizes several trials that have incorporated radioimmunotherapy to the conditioning regimen. While no comparative trials against standard radiation therapy have been done, these phase I–II trials have shown this approach to be safe with low TRM.

The Blood and Marrow Transplant Clinical Trials Network (BMT CTN) conducted a prospective multicenter randomized phase III trial (0401)^56^ comparing high-dose therapy with carmustine, etoposide, cytarabine, and melphalan (BEAM) plus rituximab vs BEAM plus conventional dose tositumomab (Bexxar) followed by autoHCT in patients with chemotherapy-sensitive relapsed DLBCL. The Bexxar/BEAM and the R/BEAM regimens produced similar 2-yr PFS and OS for patients with chemosensitive, relapsed DLBCL. No differences in engraftment or other toxicities were apparent other than an increase in mucositis with the Bexxar/BEAM regimen. No significant difference in the risk of MDS or AML was detected with the follow up available (median 25.5 months) at the time of publication.

Despite all the experience and information gathered on this disease there remains no regimen that has demonstrated superiority. Studies published in the Rituximab era appear to show less toxicity than the pioneering works of the 90’s, but no significant improvements in OS rates was demonstrated for patients submitted to HDT/ASCT.

While salvage with R-chemotherapy followed by HDT/ASCT remains a viable option, new strategies have to be taken in account for the remaining patients with relapsed/refractory aggressive lymphomas.

For the time being, allogeneic SCT and, very recently, T-replete haploidentical SCT^57^ represent the most attractive alternative. For patients who failed a previous ASCT, encouraging results have been seen with allogeneic SCT as a salvage strategy. The European Group for Blood and Marrow Transplantation (EBMT) registry published a retrospective analysis of 101 patients (ref15 Two-third of the patients received a reduced-intensity conditioning (RIC) regimen and 70% had an identical sibling donor. Outcomes at 3 years were encouraging, with a non-relapse mortality (NRM) rate of 28.2%, a relapse rate of 30%, a PFS rate of 41%, and an OS rate of 53%. Patients with a long remission after autoSCT and with sensitive disease at the time of allogeneic SCT seem to be the best candidates for this approach

## T-Cell Lymphoma

Peripheral T-Cell lymphoma (PTCL) and natural killer/T-cell lymphoma constitute a rare and very heterogeneous group of NHL. In Western countries, they account for 10% to 15% of all adult lymphomas. With the exception of anaplastic large-cell lymphoma (ALCL) positive for anaplastic lymphoma-kinase (ALK), PTCL carries a poor prognosis with low OS and DFS with conventional chemotherapy.

The most frequent T-Cell lymphoma is represented by PTCL not otherwise specified, ALCL and by angioimmunoblastic T-cell lymphoma (AITL).

Five prospective studies incorporating HDT/ASCT as part of first-line therapy have been reported. Corradini et al.^58^ published the results of two prospective phase II trials, investigating the role of high-dose sequential chemotherapy followed by ASCT in 62 patients with PTCL. Conditioning regimen consisted of mitoxantrone and melphalan or carmustine, etoposide, Ara-C and melphalan followed by PBSC autografting. In an intent-to-treat analysis, 74% completed the whole programme, whereas 16 patients did not undergo ASCT, mainly due to disease progression. At a median follow-up of 76 months, the estimated 12-year OS, DFS and EFS were 34, 55 and 30%, respectively. OS and EFS were significantly better in patients with ALK-positive ALCL, as compared with the remaining PTCL. Multivariate analysis showed that patients obtaining CR before ASCT had a statistically significant benefit in terms of OS and EFS.

Reimer et. al^59^ reported the results of a prospective, multicenter study which included 83 patients. Main subgroups were PTCL not specified (n=32) and angioimmunoblastic T-cell lymphoma (n = 27). Fifty-five (66%) of the 83 patients received transplantation. The main reason for not receiving ASCT was progressive disease. The treatment regimen consisted of four to six cycles of cyclophosphamide, doxorubicin, vincristine, and prednisone followed by mobilizing therapy with either the dexamethasone, carmustine, melphalan, etoposide, and cytarabine protocol or the etoposide, methylprednisolone, cytarabine, and cisplatin protocol and stem-cell collection. Patients in CR PR underwent myeloablative chemoradiotherapy (fractionated total-body irradiation and high-dose cyclophosphamide) and ASCT. In an intent-to-treat analysis, the overall response rate after myeloablative therapy was 66% (56% CR and 8% PR). The estimated 3-year overall and disease-free survival rates for patients in CR (calculated from CR to the date of relapse) and 3-year progression-free survival rate were 48%, 53%, and 36%, respectively.

The Nordic Lymphoma Group (NLG)^60^ conducted a large prospective phase II study in untreated systemic PTCL. Treatment-naive patients with PTCL age 18 to 67 years (median, 57 years) were included. ALK-positive ALCL was excluded. An induction regimen of six cycles of biweekly CHOEP (cyclophosphamide, doxorubicin, vincristine, etoposide, and prednisone) was administered. If in complete or partial remission, patients proceeded to consolidation with HDT/ASCT. 160 patients had histopathologically confirmed PTCL. A total of 115 underwent HDT/ASCT, with 90 in complete remission at 3 months post-transplantation. Early failures occurred in 26%. Treatment-related mortality was 4%. Consolidated 5-year OS and PFS were 51% and 44%, respectively. Best results were obtained in ALK-negative ALCL.

The Gel-Tamo Study Group investigator published their experience in 26 patients with PTCL, excluding ALK-positive ALCL^61^. Patients received 3 courses of mega-CHOP (dose-escalated CHOP) and, if the gallium scan was negative, 1 additional course followed by ASCT. Those who remained positive received 2 courses of ifosfamide and etoposide and, if in partial remission (PR) went to ASCT. After ASCT, 19 patients were in CR. Six patients were not transplanted, 5 due to progressive disease and 1 due to lethal toxicity. At two years following transplant, OS and PFS were 73% and 53%, respectively.

Mercadal et al.^62^ Reported the results of a phase II trial in 41 patients with PTCL. Induction chemotherapy included high-dose CHOP, alternating with etoposide, cisplatin, cytarabine and prednisone for a total of 6 courses. Responders received ASCT. Only 20 patients achieved complete or partial response, and 17 proceeded to transplant. With a median of follow-up of 3,2 years the PFS and OS rates at 4 years were 30% and 39% respectively.

These publications (Table 4) suggest that consolidation of CR1 with ASCT is feasible and safe. However up to one-third of patients may never receive transplant mostly due to progressive disease, thus pointing to the need for better induction regimen. The results suggest improvement in OS and DFS when compared with chemotherapy alone. In the setting of relapsed disease, ASCT has shown results comparable to those achieved in relapsed aggressive B-cell lymphoma. Long-term DFS was reported in 30% to 50% of patients, making auto-HCT effective therapy for this indication. In patients with refractory disease the outcome remains poor and other strategies are needed.^6^-^8^ HTD/ASCT is generally not recommended in this setting because relapse rates have been exceedingly high and long-term survival cannot be expected.

## Conclusions

High dose therapy followed by autologous stem cell transplantation (HDT/ASCT) together with allogeneic and haploidentical stem cell transplantation have still clear roles in the treatment armamentarium of an highly heterogeneous disease known as aggressive lymphomas.

Regarding B-cell lymphomas, the recent incorporation of Rituximab into virtually all first-line therapies of all B-cell lymphomas resulted in major improvements of conventional therapy. Even if a consensus is a missing due to the fact that results of the studies incorporating HDT/ASCT in the frontline therapy of aggressive B-cell lymphoma are contradictory, a significant proportion of high risk (high aaIPI) may still benefit from HDT/ASCT, at least in terms of a longer disease-free survival.

On the other hand, at the present time, patients with relapsed or refractory B-cell lymphoma are more difficult to salvage than in the pre-rituximab era. While salvage with R-chemotherapy followed by HDT/ASCT remains a viable option in the Rituximab era, it is surely not the best. Results from clinical trials are disappointing for relapsed patients, and thus novel strategies, which can enhance the anti-lymphoma effect, at the same time reducing toxicity need to be exploited with the aim of improving the outcome of HDT/ASCT in aggressive NHL. Studies testing novel high-dose strategies prior to ASCT and studies comparing the efficacy of alloSCT and HDT/ASCT are warranted, and some of them are currently on their way.

For patients with T-cell lymphoma, no major progress has been made over the last decade and phase III studies comparing chemotherapy to novel agents or combining them are still ongoing. However, no magic bullet such as Rituximab for B-cell lymphoma seems to stand in the landscape of T-cell lymphomas. In this regard, even if the results of some studies support the use of HDT/ASCT for patients with T-cell lymphoma, showing that a small proportion of patients with T-cell lymphoma may survive several years without disease after HDT/ASCT, we think that allogeneic SCT will play an increasing role for all patients with T-cell lymphoma, including high-risk patients needing first-line therapy.

## Figures and Tables

**Table 1:** Phase III trials of HDT/ASCT in unfavorable non-Hodgkin lymphoma patients

Author	Year	Patients (n°)	DLCL (%)	aaIPI ≥2(%)	PFS/EFS (%)	*P*	OS(%)	*P*
Gianni [1]	1997	98	88 91	74 94	7y:49 7y:76	0.001	7y:55 7y:81	0.09
Santini [2]	1998	124	72 77	59 54	6y:48 6y:60	N.S.	6y:65 6y:65	N.S.
Gisselbrecht [3]	2002	270	62.5 60	97 99	5y:52 5y:39	0.01	5y:60 5y:46	0.007
Kaiser [4]	2002	312	61 58	75 73	3y:49 3y:59	N.S.	3y:63 3y:62	N.S.
Martelli [5]	2003	150	84 78	100 100	5y:49 5y:61	N.S.	5y:65 5y:64	N.S.
Olivieri [6]	2005	222	78 75	68 72	7y:44.9 7y:40.9	N.S.	7y:60 7y:57	N.S.
Vitolo [7]	2005	126	90 80	80 87	6y:48 6y:45	N.S.	6y:63 6y:49	N.S.
Betticher [8]	2006	129	69 76	88 72	3y:33 3y:39	N.S.	3y:53 3y:46	N.S.
Haioun [9]	2000	451	60 55	100 100	8y:55 8y:39	0.02	8y:64 8y:49	0.04
Baldisserra [10]	2006	56	N.R.	100 100	5y:47 5Y:30	N.S.	5y:47 5y:40	N.S.
Klui-Nelemans [11]	2001	311	50 58	31 29	5y:61 5y:56	N.S	5y:68 5Y:77	N.S.
Milpied [12]	2004	197	74 76	56 49	5y:55 5y:37	0.037	5y:74 5y:44	0.001

Legend. DLCL: diffuse large cell lymphoma, aaIPI: adjusted-age International Prognostic Index, PFS: progression-free survival, EFS: event-free survival, OS: overall survival, NS: not significant.

**Table 2:** Studies of HDT/ASCT in unfavorable DLBCL patients

Author	Year	Patients (n°)	Age	DLCL (%)	aaIPI≥2 (%)	PFS/EFS (%)	OS (%)
Tarella [14]	2007	112	18–66	79	100	4y:73	4y:76
Vitolo [15]	2009	97	19–60	86	100	4y:73	4y:80
Fitoussi [16]	2011	209	18–60	N.R.	100	4y:76	4y:78
Stiff [17]	2011	370	18–65	89	100	2y:72	N.S.
Vitolo [18]	2011	412	18–65	100	100	2y:71	N.S.
Le Gouill [20]	2011	340	18–60	100	N.R.	3y:41	N.S.
Schmitz [19]	2011	306	18–60	100	100	3y:69.8	3y:77

Legend. DLCL: diffuse large cell lymphoma, aaIPI: adjusted-age International Prognostic Index, PFS: progression-free survival, EFS: event-free survival, OS: overall survival, NS: not significant.

**Table 3:** Radioimmunotherapy in conventional or high dose as part of conditioning regimens for autologous transplantation in lymphoma

Study	Patients (n°)	Histology	RIT	Regimen	PFS (%)	OS (%)
**Standard-dose RIT + Chemotherapy**						
Vose et al [46]	23	Aggressive	^131^I	BEAM	3y: 39	3y: 55
Khouri et al [47]	26	Various	^90^Y	BEAM	3y: 83	3y: 92
Vose et al [48]	40	DLBCL	^131^I	BEAM	3y: 70	3y: 81
Shimoni et al [49]	23	Aggressive	^90^Y	BEAM	3y: 52	3y: 67
Shimabukuro-Vornhagen et al [50]	10	DLBCL and FL	^90^Y	BEAM	NR	NR
Krishnan et al [51]	41	Most DLBCL	^90^Y	BEAM	3y: 70	2y: 89
Decaudin et al [52]	77	Most FL	^90^Y	BEAM	2y: 63 (EFS)	2y: 97
**High-dose RIT + Chemotherapy**						
Press et al [53]	52	Various	^131^I	Cy,VP16	2y: 68	2y: 83
Nademanee et al [54]	42	Various	^90^Y	Cy,VP16	4y: 65 (DFS)	4y: 81
Winter et al [55]	44	Various	^90^Y	BEAM	3y: 43	3y: 60

Legend. RIT: radioimmunotherapy, BEAM= BCNU-etoposide-cytarabine-melphalan, DLBCL = diffuse large B-cell lymphoma, FL = follicular lymphoma, NR = not reported.

**Table 4:** Prospective series on the use of fronline ASCT in high-risk PTCL

Author	Patients (n°)	Median age (years)	ASCT (%)	ORR Pre-ASCT (%)	TRM (%)	OS (%)	PFS (%)	Follow-up (months)
Corradini et al. [58]	62 (19 ALK+)	43	74	72	4.8	34	30	76
Reimer et al. [59]	65 (No ALK+)	49	65	73	3	50	NA	10^*^
D’Amore et al. [60]	166 (No ALK+)	57	71	Not Known	4	51	44	60
Rodriguez et al. [61]	26 (No ALK+)	44	77	77	0	75	53	24*
Mercadal et al. [62]	41 (No ALK+)	47	41	59	3	39	30	47

Legend. DLCL: diffuse large cell lymphoma, aaIPI: adjusted-age International Prognostic Index, PFS: progression-free survival, EFS: event-free survival, OS: overall survival, NS: not significant.
